# Expression of p73, a novel protein related to the p53 tumour suppressor p53, and apoptosis in cholangiocellular carcinoma of the liver

**DOI:** 10.1038/sj.bjc.6690465

**Published:** 1999-06

**Authors:** A Tannapfel, K Engeland, L Weinans, A Katalinic, J Hauss, J Mössner, Ch Wittekind

**Affiliations:** Institute of Pathology, University of Leipzig, Liebigstr. 26, 04103 Leipzig, Germany; Department of Internal Medicine II, University of Leipzig; Ph.-Rosenthal-Str. 27, 04103 Leipzig, Germany; Institute of Cancer Epidemiology, University of Lübeck, St Jürgen-Ring.66, 23564 Lübeck, Germany; Department of Surgery II, University of Leipzig; Liebigstr. 20a, 04103 Leipzig, Germany

**Keywords:** cholangiocellular carcinoma, p73, p53, apoptosis, prognosis

## Abstract

p73, the first homologue of the tumour suppressor protein p53, was recently discovered on chromosome 1p36 and has been shown to induce apoptosis in a p53-like manner. The present study was performed with the aim of investigating the expression of p53, its new homologue p73 and the occurrence of apoptosis in cholangiocellular carcinoma. Protein levels of p73 were examined in 41 patients with curatively (R0-) resected cholangiocellular carcinomas with an antiserum, raised against a peptide in the N-terminal domain of p73. The incidence of mutations in the p53 gene was analysed by direct sequencing and also immunohistochemically. Apoptotic cell death was assessed using in-situ end-labelling (ISEL) technique in combination with morphological criteria. The results obtained were correlated with patient survival. Immunostaining of p73 protein was detected in 17/41 carcinomas examined (41%). The immunoreactivity was confined to the cell nucleus. In 15/41 patients (37%), mutations of the p53 gene were observed. Eleven out of these 15 patients stained also positive for p73. In contrast, out of 26 patients without any detectable p53 mutation, only six exhibited p73 immunostaining. We failed to observe a correlation between p73 expression or p53 and apoptosis within a given tumour. Survival analysis including the parameters stage and grade of disease, p73 and p53, and also apoptosis, showed that tumour stage and grade as well as p53 and p73 were significantly related to prognosis. In Cox regression survival analysis, however, only extent of primary tumour and lymph node status had an independent prognostic impact. Our results with a high prevalence of p73 within tumours harbouring mutated p53 gene suggest that p73 could compensate for p53 function. We failed to establish p73 or p53 as independent prognostic factors in cholangiocellular carcinoma of the liver. © 1999 Cancer Research Campaign


*British Journal of Cancer* (1999) **80**(7), 1069–1074
© 1999 Cancer Research Campaign
Article no. bjoc.1998.0465

### Expression of p73, a novel protein related to the p53 tumour suppressor p53, and apoptosis in cholangiocellular carcinoma of the liver

**A Tannapfel[1], K Engeland[2], L Weinans[1], A Katalinic[3], J Hauss[4], J Mössner[2] and Ch Wittekind[1]**

[1]Institute of Pathology, University of Leipzig, Liebigstr. 26, 04103 Leipzig, Germany; [2]Department of Internal Medicine II, University of Leipzig; Ph.-Rosenthal-Str. 27, 04103 Leipzig, Germany; [3]Institute of Cancer Epidemiology, University of Lübeck, St Jürgen-Ring.66, 23564 Lübeck, Germany; [4]Department of Surgery II, University of Leipzig; Liebigstr. 20a, 04103 Leipzig, Germany

**Summary** p73, the first homologue of the tumour suppressor protein p53, was recently discovered on chromosome 1p36 and has been shown to induce apoptosis in a p53-like manner. The present study was performed with the aim of investigating the expression of p53, its new homologue p73 and the occurrence of apoptosis in cholangiocellular carcinoma. Protein levels of p73 were examined in 41 patients with curatively (R0-) resected cholangiocellular carcinomas with an antiserum, raised against a peptide in the N-terminal domain of p73. The incidence of mutations in the p53 gene was analysed by direct sequencing and also immunohistochemically. Apoptotic cell death was assessed using in-situ end-labelling (ISEL) technique in combination with morphological criteria. The results obtained were correlated with patient survival. Immunostaining of p73 protein was detected in 17/41 carcinomas examined (41%). The immunoreactivity was confined to the cell nucleus. In 15/41 patients (37%), mutations of the p53 gene were observed. Eleven out of these 15 patients stained also positive for p73. In contrast, out of 26 patients without any detectable p53 mutation, only six exhibited p73 immunostaining. We failed to observe a correlation between p73 expression or p53 and apoptosis within a given tumour. Survival analysis including the parameters stage and grade of disease, p73 and p53, and also apoptosis, showed that tumour stage and grade as well as p53 and p73 were significantly related to prognosis. In Cox regression survival analysis, however, only extent of primary tumour and lymph node status had an independent prognostic impact. Our results with a high prevalence of p73 within tumours harbouring mutated p53 gene suggest that p73 could compensate for p53 function. We failed to establish p73 or p53 as independent prognostic factors in cholangiocellular carcinoma of the liver.

**Keywords:** cholangiocellular carcinoma; p73; p53; apoptosis; prognosis

Intrahepatic cholangiocarcinoma is a usually fatal malignant neoplasm originating from biliary epithelia or cholangiocytes, and constitute about 5% of primary liver cancers (Wittekind et al, 1995). To date, however, the cellular and molecular mechanisms leading to oncogenesis of cholangiocytes remain unclear. Increasing evidence exists that carcinogenesis must be understood in terms of accumulation of mutations in regulatory genes, including activation of oncogenes and inactivation or loss of tumour suppressor genes. Among the available candidates, the p53 gene has come to be known as a ‘master guardian of the genome’, because of its role in regulating of cell growth and death (Harris, 1996). Recently, a new gene with significant homology to p53 has been discovered and named p73 (Kaghad et al, 1997). This gene mapped to the short arm of chromosome 1. It has also been reported recently that p73 can – at least when overproduced – activate the transcription of p53-responsive genes and inhibit cell growth, in a p53-like manner by inducing apoptosis (Jost et al, 1997). But unlike p53, no significant mutation of the p73 has been found in human tumours. In lung cancer, activation of a silent allele and overexpression of wild type p73 has been reported recently (Mai et al, 1998; Nomoto et al, 1998).

*Received 21 September 1998*
*Revised 22 December 1998*
*Accepted 22 December 1998*

*Correspondence to:* A Tannapfel

No data describing the expression of p73 in cholangiocellular carcinomas in correlation to the mutation status of p53 have so far been reported. We therefore performed immunohistochemistry for the p73 protein expression, p53 mutation analysis and also survival analysis in patients with cholangiocellular carcinoma of the liver.

### MATERIALS AND METHODS

### Patients and tissue samples

Forty-one patients with cholangiocellular carcinoma (CCC) undergoing partial hepatectomy (segmental or lobar resection) between 1994 and 1997 were included in this retrospective study.

No patient received preoperative or adjuvant chemo- or radiotherapy. All patients underwent surgery in curative intent (R0 resections). Patients who received orthotopic liver transplantation were excluded from this study.

Each tumour was re-evaluated in regard to typing, staging and grading (WHO, 1994). Tumour typing and staging was performed using WHO and UICC (1997) criteria respectively. In addition, every tumour was examined macro- and microscopically for the presence of vascular invasion, satellites, multiplicity, inflammatory reaction, necrosis and dysplasia in the surrounding liver tissue and cirrhosis. In all cases, slides prepared from four different paraffin blocks of tissue, sampled from different tumour areas, were examined. Pathohistological data are summarized in Table 1.

**Table 1** Patients and pathohistological data

| | No. of patients | 1-year survival rate (%) (95% CI) | Median survival (95% CI) (days) | Odds ratio (crude) |
|---|---|---|---|---|
| All | 41 | 31 (17–45) | 210 (156–264) | |
| Stage | | | | |
| I | 1/41 (5%) | 100 | Patient alive (after 1596 days)[a] | |
| II | 7/41 (15%) | 100 | Seven patients alive (after 257–1323 days) | |
| IIIA | 21/41 (51%) | 24 (0–44) | 210 (134–286) | |
| IIIB | 3/41 (7%) | 0 | 80 (48–112) | |
| IVA | 9/41 (22%) | 0 | 89 (0–47) | |
| pT-category | | | | |
| 1/2 | 8/41 (20%) | 100 | All alive | |
| 3/4 | 33/41 (80%) | 15 (3–27) | 159 (111–107)[a] | |
| pN-category | | | | |
| 0 | 32/41 (78%) | 40 | 251 (215–287) | |
| 1 | 9/41 (22%) | 0 | 80 (56–104) | 8.4 (3.4–20.4) |
| Grading | | | | |
| G1 | 14/41 (34%) | 48 (21–75) | 369 | |
| G2 | 21/41 (51%) | 20 (4–36) | 156 (71–241) | 2.8 (1.2–6.5) |
| G3 | 6/41 (15%) | 17 (0–47) | 80 (0–174) | 3.7 (1.3–11.9) |
| p73-positive | | | | |
| Yes | 17/41 (41%) | 6 (0–17) | 132 (45–219) | |
| No | 24/41 (59%) | 49 (29–69) | 320 | 4.9 (2.2–10.5) |
| p53 mutation | | | | |
| Yes | 15/41 (37%) | 6 (0–18) | 120 (66–174) | |
| No | 26/41 (63%) | 45 (25–65) | 260 | 4.2 (2.0–8.9) |
| p53 wild-type *and* p73-negative | 20/41 | 60 (38–82) | >320 | |
| p73-positive *or* p53 mutation | 21/41 | 5 (0–15) | 132 (64–200) | 6.2 (2.6–14.5) |

[a]Calculation not possible (insufficient number of patients within the category).

### Specificity of the antibody

The rabbit polyclonal p73 antibody was raised against a 14 amino acid peptide in the N-terminal part of p73 in which p73 is different from p53. The amino acid sequence of the peptide is specified as follows: $NH_2$-FHLEGMTTSVMAQF-COOH. The p73 antiserum is not cross-reactive with p53. Western blot analysis was performed as described before (Tannapfel et al, 1998) using lysate from seven cholangiocellular carcinomas to show protein bands, specific for p73 and p73α (Figure 1, left). The lysate of the same tumours were used for Western blot against p53 (Figure 1, right).

### p53 sequencing

DNA was extracted according to standard procedures (Sambrook et al, 1989) from tumour and corresponding non-neoplastic liver. Inasmuch as 98% of p53 gene mutations in diverse types of cancers have been found in exons 5–9, we focused our study on these exons (Harris 1996). Each exon from 4 to 9 of the p53 gene was amplified by 35 cycles of polymerase chain reaction (PCR) using 5′-end-labelled primers and *Taq* polymerase (Perkin Elmer/Cetus, Norwalk, CT, USA). The following primers were used to amplify p53 exons 4–9: exon 4: 5′CCT GTG GGA AGC GAA AA 3′ and 5′GCA AGA AGC CCA GAC GGA AAC 3′; exon 5: 5′ TGT TCA CTT GTG CCC TGA CT 3′ and 5′CAG CC TGT CGT CTC TCC AG 3′; exon 6: 5′ TGG TTG CCC AGG GTC CCC AG 3′ and 5′ TTA ACC CTT CTT CCC AGA GA 3′; exon 7: 5′ CCC CTG CTT GCC ACA G 3′ and CTA CTC CCA ACC ACC CTT GT 3′; exon 8/9: 5′ AAG GGT GGT TGG GAG TAG A 3′ and 5′ AAA CGG CAT TTT GAG TGT TAG 3′. In difficult cases, nested PCR was used for minimizing the background during sequencing. The sequences of all primers used for amplification are available from the authors upon request. DNA sequencing of the PCR products were performed using the DNA-Sequenase-Kit (Amersham, Germany) and an automatic sequencing analyser (ABI 373; Applied Biosystems-Perkin-Elmer, Germany).

### Immunohistochemical analysis and assessment

Immunohistochemistry and in-situ end-labelling (ISEL) technique was performed according to previously published methods from tumour and corresponding non-tumourous tissue (Tannapfel et al, 1996, 1998). Apoptosis assessment was performed using the following labelling mixture after proteinase K digestion (Sigma-Aldrich Biochemicals[R], St Louis, MO, USA): 0.01 mM each of dATP, dCTP, dGTP, and fluorescein-labelled dUTP (Boehringer Mannheim[R], Germany), 5 mM magnesium chloride; 10 mM β-mercaptoethanol; 5 mg $ml^{-1}$ bovine serum albumin; and 20 units $ml^{-1}$ Klenow DNA polymerase fragment (Boehringer Mannheim[R], Germany).

For immunohistochemistry, the sections were with the primary antiserum against p73 or the p53-antibody respectively (p53:

 *© 1999 Cancer Research Campaign*

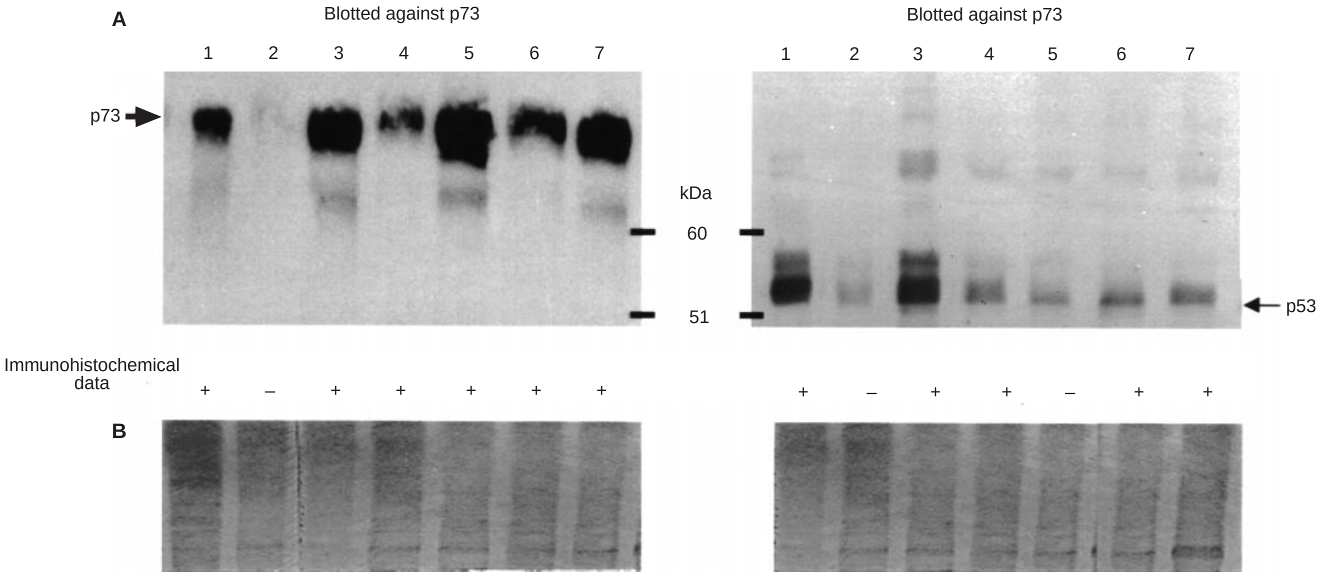

**Figure 1** Western blot (immunoblot) analysis of seven cholangiocellular carcinomas (CC) (Lanes 1–7). Immunoblot analysis of p73- (left) and p53 protein (right). (**A**) Detection of p73 and p53 protein in cholangiocellular carcinoma. A total of 50 μg cellular protein in each track was subjected to SDS-PAGE (12%) and blotted to nitrocellulose. Blots were then incubated with p73 antiserum ([left] and p53-antibody (Clone DO-7; Dakopatts, Denmark) [right] (see also Materials and Methods). In case 3, 5 and 7 an additional band for p73α was observed, as described by Khaghad et al (1997). (**B**) Identical gels stained with Coomassie blue. The reduction of size was due to the gel drying procedure. Histopathological data of the tumours examined: Lane 1: well (G1) differentiated CC, Stage IIIA; Lane 2: well (G1) differentiated CC, Stage I; Lane 3: moderately (G2) differentiated CC, Stage IIIA; Lane 4: moderately (G2) differentiated CC, Stage IIIA; Lane 5: well (G1) differentiated CC, Stage IIIB; Lane 6: moderately (G2) differentiated CC, Stage IIIA; Lane 7: moderately (G2) differentiated CC, Stage IVA

Clone DO-7, dilution 1:1000; Dakopatts[R], Denmark). Two sections from two different paraffin-embedded tumour tissue blocks were examined and scored independently by two of us in the absence of any clinical or pathological information. The positivity of the markers (ISEL-positive cells and p53-positive tumour cell nuclei) was assessed by counting an average number of 800 tumour cells, in sections of 200 cells each in four different fields of every tumour. For p73 evaluation, we scored a tumour as positive in case of at least 10% positive tumour cell nuclei. Two slides were counted in every case, leading to a total of 1600 evaluated tumour cells for each carcinoma. An eyepiece integration grid was used to ensure that cells were evaluated only once. Stained tumour cells were considered to be positive, using a light microscope (magnified 400 ×).

The intra-observer error was calculated in a preliminary examination using the same material; we found that at least 250 tumour cell nuclei needed to be assessed in order to have the results fall within the 5% of the estimated real mean with a probability of 95%. To minimize inter-observer error, all counts were performed separately. In three cases, in which conflicting numbers of positive cells were evaluated, recounting was performed to obtain a concordance of opinion.

### Statistics

Differences in frequencies between subgroups were analysed with the Kruskal–Wallis test and the Mann–Whitney *U*-test for unpaired samples. Correlation coefficients were calculated according to Pearson, and $\chi^2$ statistics were used for contingency tables. Overall observed survival functions and probabilities were estimated with the Kaplan–Meier method. The log-rank test was used to detect differences between survival curves for stratified variables. Identification of relevant prognostic factors were performed with univariate Cox regression analyses. The significance level was defined as $P < 0.05$. The median follow-up of our patients was 210 days (range: 50–1749 days). No patient was lost during follow-up.

The medical records of all 41 patients were re-examined to assess the status of disease at the closing date of the study (30 April, 1998). At this time, eight patients were still alive. All the patients who died during the follow-up period had intrahepatic and metastatic disease on their last visit to the oncological outpatient clinic. We concluded that death in these patients was related to CCC. Median survival was 210 days (95% confidence interval (CI) 156, 264 days), and the 1-year survival rate was 31% (95% CI: 17, 45).

### RESULTS

### Histopathological features

The pathological data are summarized in Table 1.

### p73 immunohistochemistry

Out of 41 tumours examined, p73 immunostaining was found in 17/41 cases (32%). Within these tumours, we generally observed a strong immunoreactivity of the tumour cell nuclei. With few exceptions, the cytoplasm was negative (Figure 2). p73 did not stain in all malignant cells of a tumour. Furthermore, the staining intensity varied within the tumour, without a predominant localization. Mitotic, apoptotic or necrotic cells were uniformly negative.

In the case of surrounding, non-neoplastic liver tissue, normal hepatocytes were occasionally seen to be slightly positive. In

© 1999 Cancer Research Campaign

**Table 2** p73 expression according to stage of disease (according to UICC) and grade (according to WHO)

| | p73-positive | p73-negative |
|---|---|---|
| Stage | | |
| I | 0/41 | 1/41 (2%) |
| II | 0/41 | 7/41 (17%) |
| IIIA | 7/41 (17%) | 14/41 (34%) |
| IIIB | 3/41 (7%) | 0/41 |
| IVA | 7/41 (17%) | 2/41 (5%) |
| Grading | | |
| G1 | 3/41 (7%) | 11/41 (27%) |
| G2 | 11/41 (27%) | 10/41 (24%) |
| G3 | 3/41 (7%) | 3/41 (7%) |

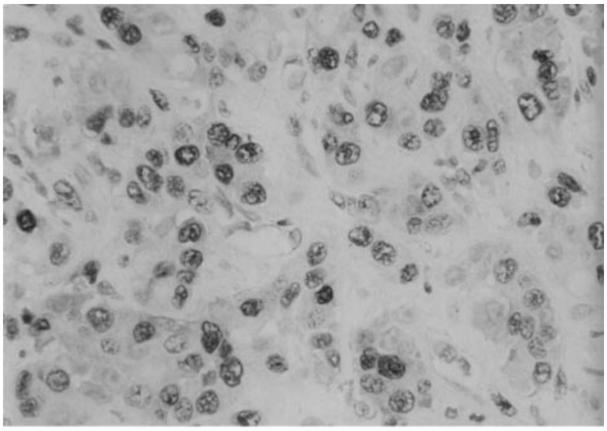

**Figure 2** p73 immunostaining of cholangiocellular carcinoma of the liver – brown reaction product within the tumour cell nuclei (DAB used as chromogen, counterstained with haematoxylin, original magnification: × 40)

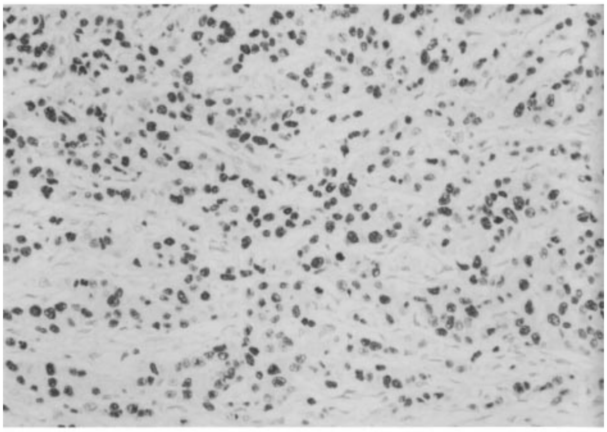

**Figure 3** p53 immunostaining of cholangiocellular carcinoma of the liver – red reaction product within the tumour cell nuclei (Fast red used as chromogen, counterstained with haematoxylin, original magnification: × 20)

contrast to the corresponding tumours, however, only very few nuclei (less than 1%) expressed p73. Bile duct epithelial cells were uniformly negative, as it was the fibrovascular stroma within the tumour. We failed to find any correlation of staining to other

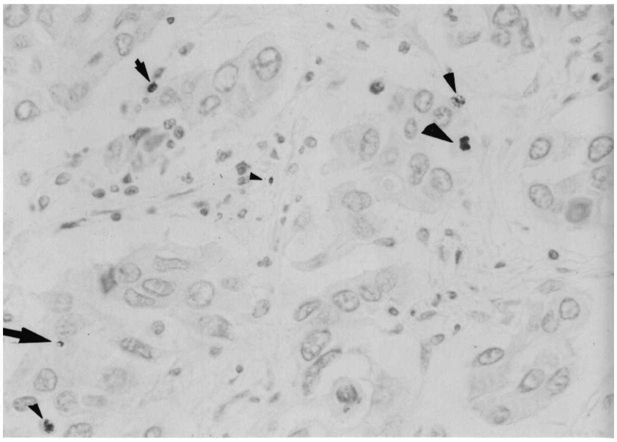

**Figure 4** Apoptotic cells (red reaction product, indicated by arrows, arrowheads), assessed by ISEL (in-situ end-labelling) technique (counterstained with haematoxylin, original magnification: × 40)

histopathological parameters examined. In particular, there were no relationships between p73 expression and tumour size, cellular differentiation or tumour stage (Table 2).

### p53 mutations

Mutations of the p53 gene were detected in 15/41 cases (36%). Point mutations of the p53 gene were detected in 14/41 cases; in one case, a microdeletion was observed. We did not find a mutation in non-cancerous tissue. In all cases, the p53 gene mutations were clustered within evolutionary conserved regions, especially in exons 4 and 7. The most common change was a C→T and G→C transition (Table 3). In two cases, silent mutations were found. There was no significant correlation between tumour stage, lymph node metastases, grade, proliferation and apoptosis and p53 mutation.

Performing immunohistochemistry, positive tumour cell nuclei, indicating a p53 mutation, were detected in 14/41 cases (34%) (Figure 3). A difference between mutation status and immunohistochemistry was observed in one tumour. In this case, a one base pair deletion was found in codon 250 of exon 7, whereas p53 immunohistochemistry was negative.

The number of p53-positive tumour cells varied from case to case, with a considerable intratumourous heterogeneity (Figure 3). Performing a semiquantitative assessment of p53 immunoreactivity (according to Sun et al, 1992; Hui et al, 1997), neither the staining intensity nor the number of positive cells correlated to the mutation pattern or to other histopathological parameters (Table 3). Non-neoplastic liver tissue was always immunohistochemically p53-negative.

### Apoptosis

Positive ISEL staining was observed in nuclei and nuclear fragments with the morphological characteristics of apoptosis (Figure 4). There was no preferential localization of apoptotic cells inside the tumour. In CCC, the apoptotic index (defined as number of apoptotic cells per 100 tumour cells) ranged between 0.1 and 1.2, with a mean of 0.56 (± 0.38). We failed to observe a statistical significant relationship between p53 mutation or p73 expression and occurrence of apoptotis within a given tumour.

*© 1999 Cancer Research Campaign*

**Table 3** p53 mutation, p73 overexpression in cholangiocellular carcinomas

| Exon | Stage | Grade | Codon | Mutation | Amino acid substitution | Immunohistochemistry | |
|---|---|---|---|---|---|---|---|
| | | | | | | p53 | p73 |
| 4 | IIIA | 1 | 125 | ACG→AAG | Thr→Lys | ++ | + |
| 4 | IIIA | 2 | 62 | GAA→GAG[a] | Glu→Glu | +++ | + |
| 4 | IIIB | 1 | 54 | TTC→TTT[a] | Phe→Phe | + | – |
| 4 | IVA | 1 | 84 | GCC→GGC | Ala→Gly | ++ | + |
| 5 | IVA | 2 | 175 | CGC→CAC | Arg→His | +++ | + |
| 5 | IVA | 2 | 178 | CAC→AAC | His→Asn | ++ | – |
| 5 | IIIA | 3 | 179 | CAT→CTT | His→Leu | ++ | + |
| 6 | IIIA | 2 | 202 | CGT→CCG | Arg→Pro | +++ | + |
| 7 | IIIB | 3 | 249 | AGG→ACG | Arg→Thr | + | + |
| 7 | IIIA | 3 | 226 | GGC→GCC | Gly→Ala | ++ | + |
| 7 | IIIB | 1 | 245 | GGC→AGC | Gly→Ser | ++ | + |
| 7 | IIIA | 3 | 250 | 1 bp del | frame shift | – | + |
| 8 | IVA | 3 | 319 | AAG→GAG | Lys→Glu | ++ | – |
| 8 | IIIA | 2 | 282 | CGG→TGG | Arg→Trp | + | – |
| 8 | IIIA | 2 | 273 | CGT→TGT | Arg→Lys | +++ | + |

[a]Silent mutations, no amino acid substitutions. p53 immunohistochemistry: positivity assessment according to (11, 13): negative (–) < 1% positive nuclei; weakly positive (+): single positive cells (10–30%); moderately positive (++): numerous positive cells (30–60%); strongly positive (+++): more than 60% positive tumour cells.

### Survival rate

The survival analysis took into account the following variables: p73 immunostaining, p53 mutational status, apoptosis, UICC tumour stage (Sobin and Wittekind, 1997), grading, vascular invasion, multiplicity, satellites, dysplasia, inflammatory reaction, necrosis, and patients' age.

As expected, UICC stage, extent of the primary tumour (pT category), presence of lymph node metastases (pN category) and histological grade of tumour differentiation, were significant prognostic parameters. Univariate analysis showed p73 and p53 to be predictors of survival. There was a significant difference in survival between patients with tumours showing p73 expression and those whose tumours did not. The median survival time for p73-positive tumours was 132 days. In contrast, tumours without detectable p73 protein had a survival time of 320 days (Figure 5). Patients with mutations of the p53 gene had a poor prognosis as well. Tumours lacking p53 mutations, and p73 expression, had a significantly longer survival time than those with mutations and/or expression (Table 1).

Multiplicity, satellites, inflammatory infiltrate, dysplasia within the non-tumourous liver and patients' age all lacked prognostic

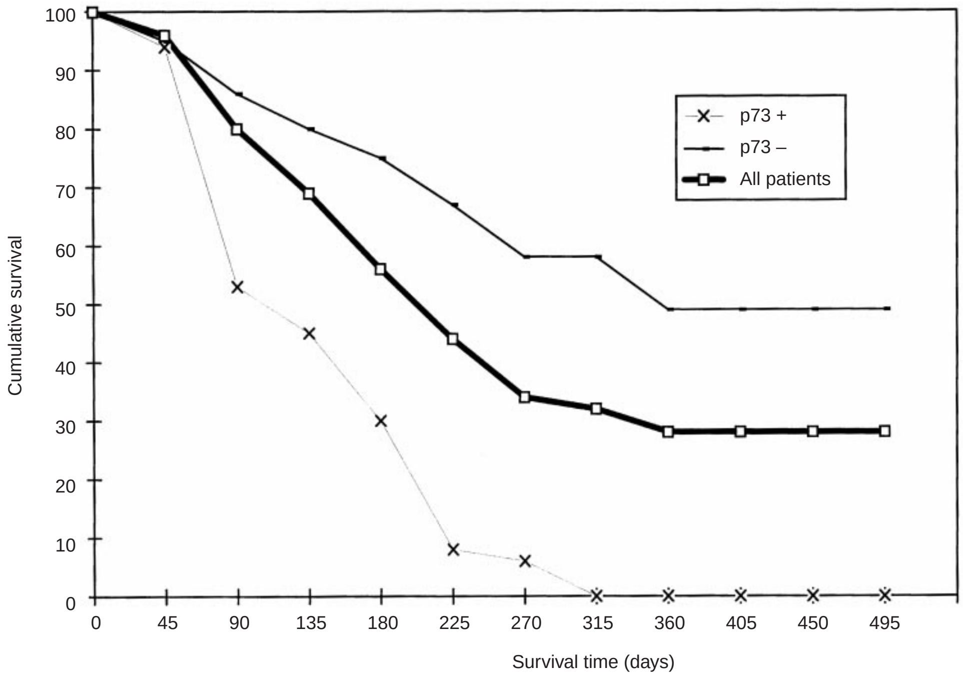

**Figure 5** Survival according to p73 immunostaining significance. The odds ratios (OR) for all factors examined are given in Table 1. Performing multivariate analysis, only pT category and pN category had an independent prognostic impact.

### DISCUSSION

Mutation of the p53 tumour suppressor gene is one of the most frequent genetic aberrations in human epithelial tumours, almost half of all primary tumours contain mutant p53 alleles. Recently, a novel gene with significant sequence homology to the DNA binding, transactivation and oligomerization domain of p53 was found (Kaghad et al, 1997). It was shown that p73 could activate transcription of p53-responsive genes and inhibit cell growth in a p53-like manner. As with wild-type p53, stable overexpression of p73 inhibits growth of a neuroblastoma cell line by inducing apoptosis (Jost et al, 1997). We therefore examined the relationship between mutational status of p53 and expression of p73 protein and the extent of apoptotic cell death in a given tumour. In cholangiocellular carcinoma, we found mutations within the p53 gene in 15/41 cases (37%). The frequency and also the mutation pattern we found were in concordance with the data from the literature (Ohashi et al, 1995). Eleven out of 15 tumours with mutations of p53 also had detectable p73. In contrast, within these 26 tumours harbouring wild-type p53, p73 positivity occurred in 6/41 cases only. These findings might suggest that mutation and disruption of p53 results in an up-regulation of p73.

In hepatocellular carcinomas with detectable mutations of the p53 gene, the expression of the cell cycle inhibitor $p21^{WAF1/CIP1}$ was found to be significantly reduced (Hui et al, 1997). The remarkable homology between p73 and p53, together with the ability of p73 to induce the expression of $p21^{WAF1/CIP1}$ (Clurman et al, 1997; Jost et al, 1997), might suggest that p73 acts, at least in part, as a transcription factor. A possible explanation for elevated p73 in cholangiocellular carcinoma, therefore, is that disruption of normal p53 function results in compensatory or deleterious up-regulation of p73 expression. Thus, either mutant p53 or reduction of p21 may trigger an increase of p73 expression. This may explain why p73 is highly expressed in tumours with p53 mutation and also why there was no detectable mutation in the p73 gene.

Another possible mechanism may be responsible for the p73 overexpression in cholangiocellular carcinomas: wild-type p53 protein, present in minute concentrations and with a short half-life in normal tissue, is not detected immunohistochemically (Sun et al, 1992). In the case of mutated p53, a protein with a longer half-life is present due to a lower rate of degradation, resulting in accumulation of p53 protein. This mechanism enables the detection of p53 in immunohistochemistry (Uhlman et al, 1994). For p73, a similar mechanism may be responsible for its accumulation in a subset of hepatocellular carcinomas.

However, we and others (Mai et al, 1998; Nomoto et al, 1998; Takahashi et al, 1998) could not find any specific somatic mutation of p73. These findings strongly suggest that p73 may play an important role in tumorigenesis through overexpression of wild-type p73 rather than as a tumour suppressor.

Our results with a median survival time for patients with cholangiocellular carcinoma are in concordance with the rates published in the literature (Ohashi et al, 1995). Long-term survival can only be achieved in case of small tumours, which can be resected curatively. Therefore, it is urgent to identify factors that can predict the prognosis of patients after tumour removal more precisely so that adjuvant therapy can be provided to different patient groups. Our results with a prognostic influence of p53 and p73 in univariate survival analysis could favour these two markers for a better prognostic assessment in an individual patient. However, we are aware that only a limited number of cases have been investigated. Therefore, the actual prognostic value of the two markers needs to be tested and proved in further, multicentric studies with an adequate number of patients and standardized treatment procedures (R0 resection), and that are within a specified stage of disease and were assessed by employing identical methods.

### REFERENCES

Clurman B and Groudine M (1997) Killer in search of a motive? *Nature* **389**: 122–123

Harris CC (1996) Molecular epidemiology of human cancer: insights from the mutational analysis of the p53 tumour-suppressor gene. *Br J Cancer* **73**: 261–269

Hui A-M, Kanai Y, Sakamoto M, Tsuda H and Hirohashi S (1997) Reduced p21WAF1/CIP1 expression and p53 mutation in hepatocellular carcinoma. *Hepatology* **25**: 575–579

Jost CA, Marin MC and Kaelin WG (1997) p73 is a human p53-related protein that can induce apoptosis. *Nature* **389**: 191–194

Kaghad M, Bonnet H, Yang A, Creancier L, Biscan JC, Valent A, Minty A, Chalon P, Lelias JM, Dumont X, Ferrara P, McKeon F and Caput D (1997) Monoallelically expressed gene related to p53 at 1p36, a region frequently deleted in neuroblastoma and other human cancers. *Cell* **90**: 809–819

Mai M, Yokomizo A, Quian C, Yang P, Tindall DJ, Smith DI and Liu W (1998) Activation of p73 silent allele in lung cancer. *Cancer Res* **58**: 2347–2349

Nomoto S, Haruki N, Kondo N, Konishi H, Takahashi T, Takahashi T and Takahashi T (1998) Search for mutations and examination of allelic expression of the p73 gene at 1p36.33 in human lung cancers. *Cancer Res* **58**: 1380–1383

Ohashi K, Nakajima Y, Kanehiro H, Tsutsumi M, Taki J, Aomatsu Y, Yoshimura A, Ko S, Kin T, Yagura K, Konishi Y and Nakano H (1995) Ki-*ras* mutations and p53 protein expression in intrahepatic cholangiocarcinomas: relation to gross tumor morphology. *Gastroenterology* **109**: 1612–1617

Orita M, Suzuki Y and Hayashi K (1989) Rapid and sensitive detection of point mutation and DNA polymorphisms using the polymerase chain reaction. *Genomics* **5**: 874–879.

Sambrook J, Fritsch EF and Maniatis T (1989) *Molecular Cloning: A Laboratory Manual*, pp E3–E4. Cold Spring Harbor, NY: Cold Spring Harbor Laboratory.

Sun XF, Carstensen JM, Zhang H, Stal O, Wingren S, Hatschek T and Nordenskjöld B (1992) Prognostic significance of cytoplasmic p53 oncoprotein in colorectal adenocarcinoma. *Lancet* **340**: 1369–1373.

Takahashi H, Ichimiya S, Nimura Y, Watanabe M, Furusato M, Wakui S, Yatani R, Aizawa S and Nakagawara A (1998) Mutation, allelotyping, and transcription analysis of the p73 gene in prostatic carcinoma. *Cancer Res* **58**: 2076–2077.

Tannapfel A, Hahn H, Katalinic A, Fietkau R, Kühn R and Wittekind Ch (1996) Prognostic value of ploidy and proliferation markers in renal cell carcinoma. *Cancer* **77**: 164–171.

Tannapfel A, Nüsslein S, Fietkau R, Katalinic A, Köckerling F and Wittekind C (1998) Apoptosis, proliferation, bax, bcl-2 and p53 status prior to and after preoperative radiochemotherapy for locally advanced rectal cancer. *Int J Radiation Oncol Biol Phys* **41**: 585–591.

Uhlman DL, Nguyen PL, Manivel C, Aeppli D, Resnick JM, Fraley EE, Zhang G and Niehans GA (1994) Association of immunostaining for p53 with metastatic progression and poor survival in patients with renal cell carcinoma. *J Natl Cancer Inst* **86**: 1470–1475.

Sobin LH and Wittekind Ch (eds) (1997) *UICC: TNM Classification of Malignant Tumors, 5th edn*. New York: Wiley-Liss

Ishak T et al (eds) (1994) *WHO: Histological Typing of Tumours of the Liver, 2nd edn*. Berlin: Springer Verlag

Wittekind C (1995) Hepatocellular carcinoma. In: *Prognostic Factors in Cancer*. Hermanek P, Gospodarowicz MK, Henson DE, Hutter RVP and Sobin LH (eds) Berlin: Springer Verlag

© 1999 Cancer Research Campaign
